# Supplementary material for: Determination of some heavy metals and their health risk in T-shirts printed for a special program
Source: PLoS One. 2022 Sep 20;17(9):e0274952. doi: 10.1371/journal.pone.0274952 (PMC9488751; doi:10.1371/journal.pone.0274952)
Supplement: Attachment — Submitted filename: Reviewers Comments 20 June 2022.docx [file pone.0274952.s001.docx]

General comments

The paper needs English editing as there are a lot of grammatical errors and typos. The paper is poorly written and that needs to be improved. Novelty is lacking and nothing much has been contributed to scientific knowledge. The paper can be considered for publication after a major revision.

Comments

1. The structure of manuscript should be changed, and a more reader-friendly formation can be achieved.
2. The abstract should have one paragraph and not three. The last paragraph of the abstract should be removed and put in the introduction. It should be re-written following the steps;
   *Introducing sentence
   *Research problem and objectives
   *Methods used in the research
   *Major findings or arguments
   *Conclusion
   This should be concise.
3. The authors should give more explanation about the novelty of this manuscript and the impact that can be given by this manuscript.
4. Some sentences are too long and messy, revising them will help the reader to understand better. The authors should avoid using ‘we’ for instance in the abstract where they wrote ‘we investigated’. It should NOT be a reported in first person.
5. The results are not well presented and its difficult for the reader to follow.
6. The correct symbol for degrees Celsius is ℃ and not C^0^ , please correct that mistake.
7. More discussion is needed.
8. Units for the concentrations need in the results section for Cobalt. There are just figures written there. The same should be done in the whole manuscript.
9. In Table 4 the heading “with skin contact’ should be replaced with “Concentration (with skin contact)”
10. In the first sentence of the discussion, the authors should check the correctness of the statement. Is red or black T-shirt which has a higher concentration.
11. The referencing in the text should be consistent. Change [17, 18 and 19] to [17-19].
12. The conclusion should be improved.
