# Supplementary material for: Determination of some heavy metals and their health risk in T-shirts printed for a special program
Source: PLoS One. 2022 Sep 20;17(9):e0274952. doi: 10.1371/journal.pone.0274952 (PMC9488751; doi:10.1371/journal.pone.0274952)
Supplement: Attachment — Submitted filename: response to reviewer.docx [file pone.0274952.s003.docx]

Comments

1. In the introduction second sentence, change the word ‘peoples’ to people. the ‘s’ should be removed. **Corrected**
2. Third paragraph of the introduction, Chromium VI should be written as Chromium (VI), the brackets are missing. **Corrected**
3. Last sentence of the third paragraph of the introduction, the word ‘especial’ should be ‘special’ **Corrected**
4. Under the results section, the first sentence should be completely removed, it has been repeated several times. The reader by now knows that AAS was used. **Corrected**
5. Under ‘method detection limit and limit of quantification’ the space between the words FOR and Blank should be removed. **Corrected**
6. Under the discussion section, the word represent should be present. **Corrected**
7. Authors should avoid putting references in the conclusion since they are concluding their own findings. **Corrected**
8. The referencing style is not the same. For instance, in some references, journal names are written in full whereas others are abbreviated. **Corrected**
